# Beyond Antibodies: Development of a Novel Protein Scaffold Based on Human Chaperonin 10

**DOI:** 10.1038/srep37348

**Published:** 2016-11-22

**Authors:** Abdulkarim M. Alsultan, David Y. Chin, Christopher B. Howard, Christopher J. de Bakker, Martina L. Jones, Stephen M. Mahler

**Affiliations:** 1 Australian Institute for Bioengineering and Nanotechnology (AIBN), University of Queensland (UQ), Brisbane, QLD 4072, Australia; 2 Centre for Advanced Imaging, University of Queensland (UQ), Brisbane, QLD 4072, Australia; 3 Australian Research Council Training Centre for Biopharmaceutical Innovation, University of Queensland (UQ), Brisbane, QLD 4072, Australia; 4 School of Chemical Engineering, University of Queensland (UQ), Brisbane, QLD 4072, Australia

## Abstract

Human Chaperonin 10 (hCpn10) was utilised as a novel scaffold for presenting peptides of therapeutic and diagnostic significance. Molecular dynamic simulations and protein sizing analyses identified a peptide linker (P1) optimal for the formation of the quarternary hCpn10 heptamer structure. hCpn10 scaffold displaying peptides targeting Factor VIIa (CE76_-P1_) and CD44 (CP7) were expressed in *E. coli*. Functional studies of CE76_-P1_ indicated nanomolar affinity for Factor VIIa (3 nM) similar to the E-76 peptide (6 nM), with undetectable binding to Factor X. CE76_-P1_ was a potent inhibitor of FX activity (via inhibition of Factor VIIa) and prolonged clot formation 4 times longer than achieved by E-76 peptide as determined by prothrombin time (PT) assays. This improvement in clotting function by CE76_-P1_, highlights the advantages of a heptamer-based scaffold for improving avidity by multiple peptide presentation. In another example of hCPn10 utility as a scaffold, CP7 bound to native CD44 overexpressed on cancer cells and bound rCD44 with high affinity (K_*D*_ 9.6 nM). The ability to present various peptides through substitution of the hCpn10 mobile loop demonstrates its utility as a novel protein scaffold.

Monoclonal antibodies (mAbs) are widely used in the life sciences as diagnostic reagents and biologic medicines. mAbs are natural protein scaffolds, which display multiple peptide loops (i.e. complementarity determining regions or CDRs), forming an extended interface that enables high affinity recognition of target antigens. There is a plethora of other molecular entities in nature with differing protein backbones that also display loops with various functionalities, and there is global interest in the development of these non-antibody binding entities, through presentation of peptides within these molecular scaffolds (Fig. 1a–d). These include scaffolds used for cancer therapy such as the Affibody^1^ targeting EGFR, DARPin^2^ targeting HER2, Monobody^3^ (of fibronectin type III) targeting VEGFR-2 for Non-Hodgkin’s lymphoma and an Anticalin^4^ (derived from lipocalins) targeting VEGF in solid tumours. An Affibody and DARPin targeting EGFR-1^5^ and HER2^6^, respectively, have also been utilised as diagnostics for tumor localisation.

Here we report the development of a novel protein scaffold based on human Chaperonin 10 (hCpn10), an essential oligomeric protein that assists in folding of translated polypeptides or refolding of denatured eukaryotic proteins^7^. hCpn10 is a homo-oligomer composed of seven subunits (Fig. 1e). Each monomer consists of β-barrel core structure that is flanked by two flexible peptide loops, known as the β-hairpin and mobile loop. The mobile loop is highly flexible in structure and is known to play a crucial role in interacting with Cpn60^8^. In this study, the mobile loop peptide was substituted with the anticoagulant peptide E-76 (binds extrinsic coagulation Factor VIIa (FVIIa)^9^ and peptide P7 which binds the cancer cell surface marker CD44^10^. Molecular dynamic (MD) modelling was used to engineer several variants of the hCpn10-E76 scaffold (hereon termed CE76) to facilitate formation of the hCpn10 native heptameric quarternary structure (Fig. 2). hCpn10 scaffold displaying seven copies of these peptides were evaluated for improved target binding as a result of imparted avidity. CE76 scaffolds were also evaluated for anti-coagulant activity compared to free peptide. Similarly, the binding of the hCpn10-P7 (named CP7) scaffold to cancer cells was evaluated as a diagnostic tool.

## Results

### Molecular dynamic (MD) simulations

MD simulations were performed to compare the structure of hCpn10 with CE76 scaffold designs. MD simulations indicated that hCpn10 is a stable molecule with flexible β-hairpin and mobile loops which do not impede heptamer formation (Fig. 2). The cluster of residues, namely, *Arg19 Ser20 Ala21 (RSA*) and *Gly38 Lys39 Val40 (GKV*) at *N*- and *C-*termini, respectively of mobile loop, packed very tightly during the simulations (Fig. 2). Also, a second possible packing scheme was observed during the MD simulations where the lysine residue (*Lys27* and *Lys35*) side chains pointed away from the loop and the polar interactions stabilised the overall extended structure of two β-strands from the molecule β-core.

The simulations indicated that CE76 formed a stable monomer but heptamer assembly was disrupted (Fig. 2). The mobile loop, which contained the E-76 sequence (*ALCDDPRVDRWYCQFVEG*) and a disulphide bond between *Cys23* and *Cys33* residues, demonstrated numerous conformational changes from the initial model whereby the E-76 loop contacted the surface of the core protein formed by the β-barrel like structure. This clearly indicated that the E-76 peptide loop may block interactions between different monomers and therefore, heptamer assembly (Fig. 2b). A number of critical interactions may also occur within the E-76 loop where polar residues *Arg30* and *Asp24* interacted with *Glu74* of the β-core, and residue *Glu37* interacted with *Lys79* of the β-core. In addition, weak hydrophobic interactions may occur between residues *Val100, Leu71*, and *Gln42* of the β-core and the CE76. Due to the flexible nature of the E-76 loop, collision occurred more frequently with β-strands 4 and 5 within the β-core of the CE76. In addition, an observed distortion on the CE76, relative to hCpn10, occurred at the dimerisation interfaces which may also affect heptamer assembly.

Subsequently, a second MD simulation was conducted on modified CE76 to address the stability issues. Several peptide-linkers were inserted (Fig. 2e) at the *N*- and *C*-junctures of the mobile loop domain (between *Ala21* and *Gly38*) with the aim of stabilising the mobile loop regions by preventing any interference with the subunit interface of hCpn10. The inserted linkers P1 and P2 were enriched with *Ala*/*Pro* residues and the E5 linker incorporated five native residues at both loop termini (Fig. 2). The key aim of using these linkers was to apply a certain degree of rigidity in the inserted peptides, to protect the cluster motif “*RSA*/*GKV*”, and to prevent interaction with the β-barrel core of hCpn10 protein.

The rationale for inserting linkers with multiple proline residues was to impose some constraint and thereby reduce the entropic rate of the inserted non-native peptides. Prolines provide rigidity to the polypeptide chain by imposing certain torsion angles on the segment of protein structure and have been found to play crucial roles in protein folding^11^,^12^.

For CE76_-P1_, the E-76 peptide was separated from the β-barrel core (hCpn10) by an *Ala-Ala-Pro* with a single proline residue in each of the *N-* and *C*-terminus, while for CE76_-P2_ two consecutive proline residues were utilised. MD simulations indicated that the *C-*terminus of both proline linker variants had only a slight interaction with the E-76 loop which in turn did not destabilise the β-core and did not impede heptamer formation. Importantly the modelling demonstrated the peptide linker modifications to CE76 created a peptide display scaffold with similar properties to the wildtype hCpn10. For the design of the CP7 scaffold targeting CD44, *Ala-Ala-Pro* linkers were incorporated at each end of the P7 peptide (Fig. 2e).

### Protein size analysis

To evaluate the theoretical predictions of the MD simulations, hCpn10 and its variant scaffolds were expressed, purified and analysed by size exclusion HPLC (SE-HPLC). The SE-HPLC analyses confirmed the results of the MD simulations, and indicated that the linker modifications, namely the inclusion of peptide linkers P1 and P2 improved scaffold assembly (Fig. 3). CE76_-P1_ was the optimal scaffold, forming stable heptamers with no higher order structures similar to the wildtype hCpn10 (Fig. 3). CE76 and the other CE76 variants produced heptamers but also had multimeric structures present. Based on the HPLC data CE76_-P1_ was selected as the candidate scaffold for E-76 bioactivity assays.

### Protein-Protein Binding

CE76_-P1_ bound specifically to recombinant FVIIa (rFVIIa) by ELISA, with a limit of detection of 1 nM and at a similar level to commercial anti-human FVIIa mAb and E76 peptide (Fig. 4a). The wildtype hCpn10 lacking the E76 peptide did not bind to rFVIIa. The binding affinity of CE76_-P1_ for rFVIIa was assessed *in vitro* using biolayer interferometry (BLI) (Fig. 4b). Scaffold CE76_-P1_ bound specifically to rFVIIa but did not bind to rFX. The binding affinity of CE76_-P1_ for rFVIIa target (K_*D*_ 3.0 nM) was similar to that of CE76_-P2_ (K_*D*_ 4.7 nM) and CE76_-E5_ (K_*D*_ 5.5 nM) and 2 fold higher than that of control E-76 peptide (K_*D*_ 6.2 nM).

Binding assays also demonstrated there was no binding of hCpn10 to rFVIIa and no binding of CE76_-P1_ to rFX. Importantly, in all detection methods, CE76 required the presence of Ca^+2^ ions in order to enable the binding to rFVIIa, although TF (Tissue Factors) was not as crucial. Furthermore, the CE76_-P1_ did not hinder the interaction between TF to rFVIIa due to the interaction of CE76_-P1_ with the exosite of FVIIa.

### Anti-coagulation activities of CE76

Further assays were conducted to determine the functional activity of CE76_-P1_, which was the CE76 scaffold with highest affinity for rFVIIa. CE76_-P1_ was a potent inhibitor of FX activity with a calculated median inhibitory concentration (IC_50_) of 1.5 ± 0.64 nM of FX that was equivalent in value to the E-76 peptide (Fig. 4c). The maximum inhibitory concentrations varied where 95% of FX activity was inhibited by CE76_-P1_ relative to E-76, which inhibited 90% of FX activity. The ability of CE76_-P1_ to inhibit the amidolytic activity, which determines FVIIa activity, was also investigated and measured using the small chromogenic substrate Chromozym *t*-PA. This in turn gives further insight into assessing whether the binding of CE76_-P1_ could affect the enzymatic active site, of the active serine domain, of FVIIa. CE76_-P1_ was a potent inhibitor of amidolytic activity, hydrolysing the small protein substrate, in the presence of TF with an IC_50_ 6.8 ± 0.55 nM, and without TF with an IC_50_ 8.4 ± 0.6 nM (Fig. 4c). This indicates that the inhibitory activity of CE76_-P1_ works independently from the TF-mediated triggering of the extrinsic clotting pathway.

The amidolytic activity assays have a larger dynamic range since the maximal inhibition by CE76_-P1_ at saturating concentrations was only 60% instead of 95% as found in the FX activation assays. Furthermore, the maximal inhibition of amidolytic activity at saturating concentrations by CE76_-P1_ of TF-FVIIa and FVIIa-only were determined to be 60 and 62% inhibitions, respectively (Fig. 4c). This indicates that TF had no substantial effect when using the activated form of FVIIa, nor does the CE76_-P1_ obstruct the TF binding to FVIIa.

The anticoagulant efficacies of CE76_-P1_ were assessed in further detail using citrated human plasma and two common clotting approaches, namely, a prothrombin time (PT) and activated partial thromboplastin time (aPTT). The aim of this study was also focussed on examining the anticoagulant properties of the newly designed CE76 through PT assays in citrated human plasma. Remarkably, the CE76_-P1_ specifically prolonged the TF-dependent coagulation pathway 7-fold over normal clotting time as determined by the PT assays (Fig. 4d), and thus potently inhibited FX activation. Furthermore, CE76_-P1_ illustrated its capacity to prolong clot formation in the PT-coagulation assays in a dose-dependent manner (Fig. 4d). In comparison, the maximal PT fold-prolongation for the control E-76 peptide plateaued at a 3-fold over normal clotting time, which was significantly lower than that of CE76_-P1_ (Fig. 4d). CE76_-P1_ prolonged clot formation 4 fold longer than E-76 peptide as determined by PT assays (Fig. 4d). Both CE76_-P1_ and E-76 peptide had no effect on clotting as determined by aPTT assays. This implied that CE76 and E-76 peptide had specific interactions with the FVII/FVIIa of the extrinsic coagulation pathway only.

### CP7 binding to CD44 on cancer cells

The binding of scaffold CP7 to MDA-MB-468 breast cancer cells which overexpress CD44 was determined by immunofluorescence. CP7 interaction with endogenous CD44 was confirmed by probing the complex with secondary rabbit anti-human Cpn10 antibody conjugated to FITC (Fig. 5). There was no binding of native hCpn10 to CD44. In order to compare with the two positive controls, i.e. P7 peptide and anti-human CD44 antibody, the cells were incubated with *His*-tagged P7-peptide followed by FITC-mouse anti-His (*C*-terminus), while monoclonal mouse anti-human CD44 antibody was detected by secondary Alexa Fluor 488-rabbit anti-mouse antibody. Both controls were visualised by fluorescence microscopy (Fig. 5). The binding of CP7 to CD44 on cells was also detected directly using florescence microscopy to visualise fluorescein DyLight 488 directly conjugated to CP7 (named 488-CP7) (Fig. 5).

CP7 binding to CD44 on MDA-MB-468 cells was also assessed using flow cytometry and demonstrated that 488-CP7 and unlabelled CP7 (binding detected by FITC secondary) both had a strong binding affinity toward MDA-MB-468 cells (Fig. 5). BLI also demonstrated that the CP7 scaffold bound specifically to rCD44 (K_*D*_ 9.6 nM) but did not bind to rCD24 (data not shown).

## Discussion

In this study hCpn10 was utilised as a novel protein scaffold for the display of peptides targeting Factor VIIa and the cancer marker CD44. hCpn10 is a homo-oligomer composed of seven subunits. Each monomer consists of a β-hairpin roof loop and a longer flexible mobile loop that are interconnected by a structurally rigid hydrophobic β-barrel core^13^. We hypothesized that the loop like structures of hCpn10, particular the mobile loop could be exploited for the display of peptides for diagnostic and therapeutic applications.

The ability of Cpn10 to accommodate changes in net charge, whilst maintaining or improving target binding through avidity effects, is critical for the application and utility of hCpn10 as a useful scaffold. The E-76 peptide was the ideal test candidate for peptide display through substitution of the Cpn10 mobile loop. Incorporation of E-76 peptide dramatically changed the isoelectric point (pI) of the protein (pI 8.91 to 6.47). MD simulations proved a valuable tool for Cpn10 scaffold design, determining peptide design considerations in the mobile loop that favour optimal heptamer formation.

MD simulations suggested that interaction of the substituted E-76 peptide with the core of Cpn10 disrupted heptamer formation. The insertion of specific proline linkers were required to stabilize the highly polar E-76 loop and prevent interactions with the protein core. The CE76_-P1_ and CE76_-P2_ cores were more stable and no significant distortions were observed with critical β-strands. In contrast, the CE76_-E5_ scaffold design with the E-76 loop further downstream of the mobile loop did not prevent interactions with the protein β-core. This suggests that the rigidity of the flanking proline residues is integral for constraint of the peptide movements and prevention of core interactions. The predictions from the MD simulations correlated closely with protein size analysis. hCpn10 and CE76_-P1_ formed heptamers only whereas the other scaffolds formed heptamers and higher order multimers.

The formation of a heptamer by CE76_-P1_ facilitated the display of seven peptide copies on the one molecule for improved avidity to target molecules. The E-76 peptide, displayed in the Cpn10 heptamer was able to prolong blood clot formation 4 times longer than achieved by free E-76 peptide as determined by PT assays. In addition to the avidity effect it is also possible that the peptide is presented in the scaffold in a differently folded conformation that improves activity. There is also potential for free-peptide to be more readily degraded by proteases, lowering peptide activity, however activity was comparable to another study^9^. In addition to Cpn10, the benefits of peptide display on heptamers has been demonstrated by fusing an oligomerisation domain to the Mycobacterium tuberculosis antigen 85A^14^. In this example the presentation of the 85A antigen in heptamer form improved molecule activity by enhancing immunogenicity, specifically the induction of T cell responses in both mice and non-human primate studies.

To confirm the versatility of hCpn10 as a display scaffold, the P7 peptide with high affinity for cancer biomarker CD44^10^ was incorporated into the mobile loop of hCpn10, generating CP7 which was tested as a potential diagnostic probe for early stage cancers. The proline linker modifications adopted for CE76_-P1_ were used for CP7. CP7 was produced as a soluble heptameric protein and binding studies indicated higher affinity for rCD44 compared to P7 peptide alone and no binding to rCD24.

In addition to the flexible loops for peptide display, Cpn10 is structurally stable, heat resistant and one of few proteins that can fold reversibly *in vitro* despite the introduction of various mutations^15^,^16^. Cpn10 assembles into a heptamer through strong hydrogen bonds between the β-barrels of contiguous monomers. It is the interactions between the antiparallel pairings of the β-strand interface of each monomer, and the inter-protein interactions that govern over 75% of the Cpn10 heptamer configuration stability^17^,^18^. It is these characteristics that make Cpn10 similar to other non-antibody scaffolds such as Affibodies and DARPins, which also have structures that are thermostable, capable of tolerating modification and able to express multiple binding sites. For example Affibodies have an alpha helix bundle that can tolerate modifications and can independently fold when incorporated in fusion proteins^19^. Head to tail fusions of Affibodies with the same target specificity improves binding avidity^20^, in a similar fashion to Cpn10 display of seven peptide copies on the one heptameric molecule. Head to tail fusions of Affibodies binding different targets can also be utilised to create multi-specific molecules^20^. DARPins have an alpha helix bundle with beta turn repeat units for target binding, whereby 4–5 repeats can be presented whilst maintaining thermodynamic stability^21^, which is similar to the Cpn10 heptamer concept. DARPins are also stable when expressed as fusion proteins enabling potential multi-specific molecules to be displayed.

The structural integrity of Cpn10 scaffolds suggests the potential for development of heptamer fusions. In addition, there is potential to use MD simulations to model the roof loop as an additional site for the presentation of peptides which would enable the presentation of 14 peptides on Cpn10. Other advantages of hCpn10 as a scaffold are that it is easily expressed in *E. coli*, is resistant to degradation, has a low immunogenicity and has been safely used in human clinical trials^22^. This is comparable to other non-antibody molecular scaffolds, an example being the DARPins which are stable in human serum and proven in clinical settings^23^.

The discovery of other molecular entities that are capable of behaving like antibodies in “beyond antibody” approaches has opened up a new area in protein therapeutics and diagnostics, and has led to the development of novel intellectual property. The research outcomes presented in this study have made a valuable contribution in the area of molecular scaffolds, and has indeed shown that human Cpn10 is another molecular scaffold capable of presenting and displaying peptides in a fashion that allows binding of the peptides to target proteins.

## Methods

### Homology modelling

**Figure 1:**
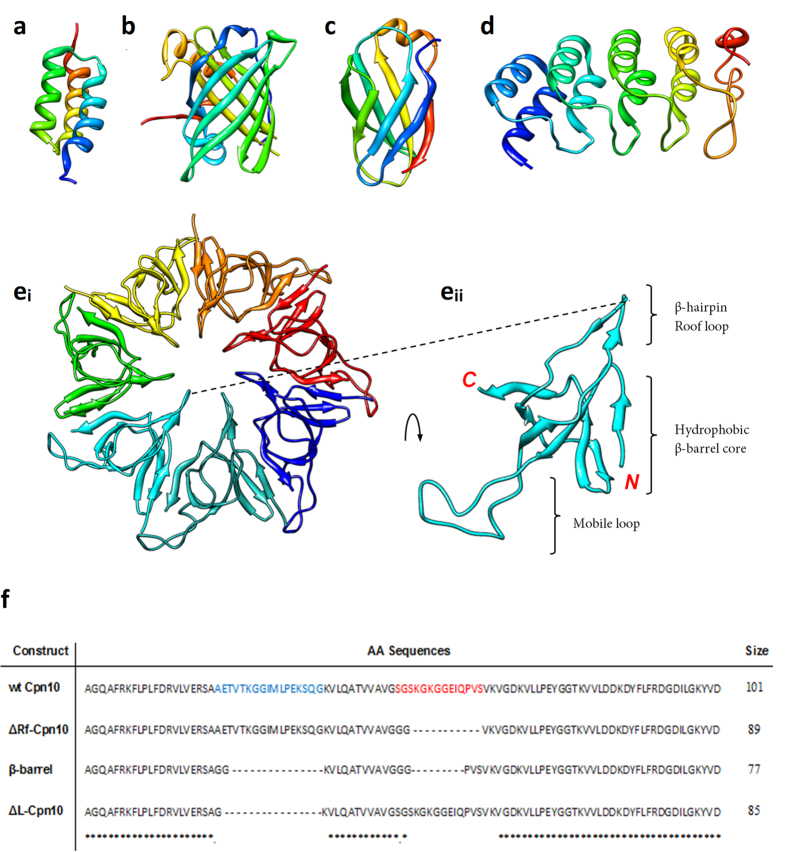
Non-antibody molecular scaffolds. Schematic representations of protein backbones used as scaffolds for generating protein-binding agents: (**a**) α-helix bundle, Affibody (PDB ID. 2B89); (**b**) β-barrel & loop, Anticalin (PDB ID. 1LNM) of lipocalin; (**c**) β-sandwich & loop, Fibronectin (FN3) (PDB ID. 1TTG); and (**d**) α-helix bundle & β-turn repeat unit, DARPin (PDB ID. 2BKK) of ankyrin repeat protein. (**e**) Loops, Chaperonin10 (Cpn10) (PDB ID. 1WE3) in full heptamer oligomer (e_i_) and in monomer (e_ii_), illustrating the mobile loop domain. All modelled by UCSF Chimera 1.5.3. (**f**) Primary sequence of Cpn10 showing residues forming mobile loop (blue) and roof (red) structures. The primary sequence of structural mutants have also been depicted including Cpn10 without the roof loop (∆Rf-Cpn10), Cpn10 without the roof or mobile loops (β-barrel) and Cpn10 without the mobile loop (∆L-Cpn10).

**Figure 2:**
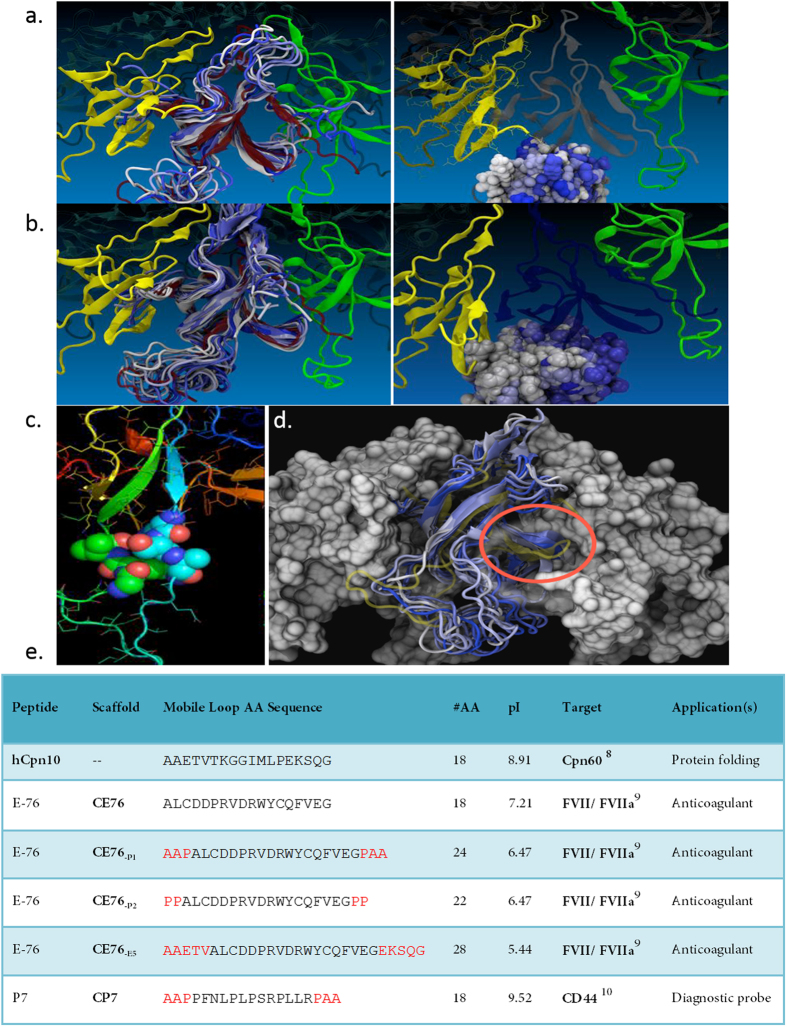
MD simulations. (**a**) Snapshots of hCpn10 over duration of 1 ns at 310 K. (left) overlaid snapshots of MD simulations of the hCpn10 monomer in ribbon representation coloured from white to dark blue (right) overlaid snapshots of atoms of hCpn10 mobile loop shown as space-filling model. (**b**) Snapshots of CE76 over duration of 1 ns at 310 K. (left) overlaid snapshots of MD simulations of the CE76 monomer in ribbon representation coloured from white to dark blue (right) overlaid snapshots of atoms of CE76 mobile loop shown as space-filling model. All snapshots show monomers over 1 ns MD simulation at 310 K, in 50 ps intervals and coloured from white (350 ps) to dark blue (700 ps), then overlaid post-MD simulations on top of the static model of the hCpn10 heptamer. (**c**) Cluster motif *RSA*/*GKV* required for β-barrel core stabilization. Cluster motif to stabilise hCpn10 protein core shows “*RSA*” and “*GKV*” as space-filled models and coloured blue and green, respectively, with strong and tight packing for β-core stabilisation. (**d**) Snapshots of MD simulations of post-modified CE76_-P1_ over duration of 1 ns at 310 K. Snapshots shown CE76_-P1_ in ribbon representation and overlaid onto the hypothetical hCpn10 structure (yellow), the location in a cut-out of the heptamer to estimate the ability of CE76_-P1_ to form heptamer. Red ellipsoid illustrates β-barrel core (β-strands 4 and 5). The series snapshots overlaid in 10 ps intervals from the last 100 ps of the calculation, and coloured from light to dark blue. (**e**) hCpn10 based display scaffolds. Scaffolds of hCpn10 based on the substitution of native mobile loop residues with E-76 and P7 peptides. The linkers for heptamer stability designed from molecular dynamic simulations are indicated in red.

**Figure 3:**
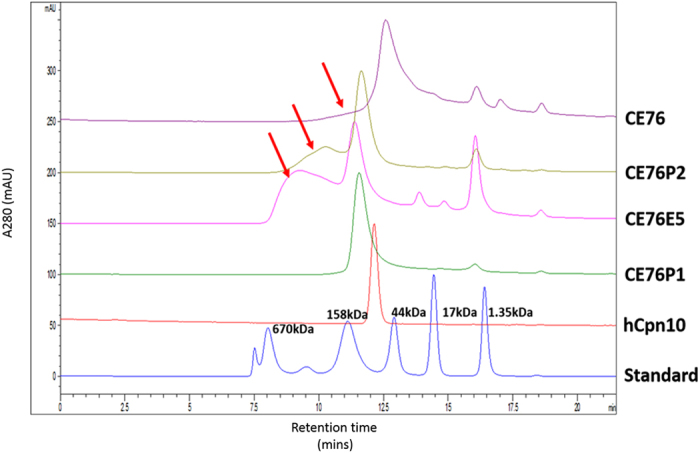
HPLC-size exclusion stack chromatograms of hCpn10 and CE76 variants. Elution profiles of hCpn10; CE76_-P1_; CE76_-E5_; CE76_-P2_; and CE76, respectively. Elution is represented on x-axis in minutes and y-axis is protein measurements at absorbance 280 nm. hCpn10 has a theoretical Mwt of 70 kDa, but has a predicted Mwt of 90 kDa by size exclusion due to its conformation and migration in chromatography. CE76 has similar elution time to hCpn10 as both have 18 residue mobile loop. The other CE76 variants have a shorter elution time than CE76 and hCpn10 as a result of increased mobile loop of 24–28 residues. CE76, CE76_-P2_ and CE76_-E5_ variants have multimeric species in addition to the main peak indicated by red arrows. CE76_-P1_ has no multimers. The column was calibrated with BioRad reference protein standard: Thyroglobulin (670 kDa); Ovalbumin (44 kDa); γ-Globulin (158 kDa); Myoglobin (17 kDa); and Vitamin B12 (1.35 kDa); respectively.

**Figure 4:**
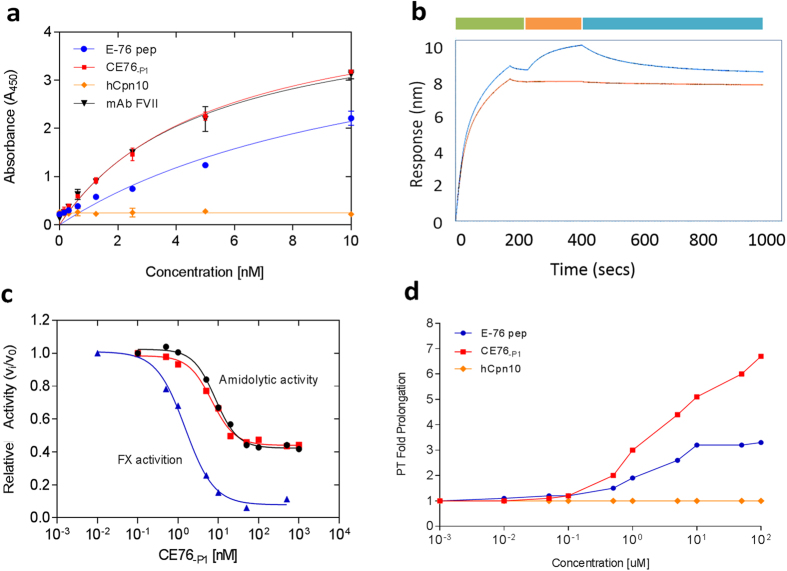
CE76_-P1_ anticoagulant *in vitro* bioassays. (**a**) Binding to immobilised rFVIIa by ELISA. Data show absorbance (A_450_) *versus* concentration (nM) of the applied peptides/proteins, with mean ± standard error (n = 3); curves fitted by nonlinear regression. (**b**) BLI sensorgram measuring binding kinetics to immobilised rFVIIa. Binding is measured as response (nm) over time (sec) of the assay. Blue curve represents binding of CE76_-P1_ and red curve is binding of hCpn10. Green block represents immobilisation of rFVIIa onto Ni-NTA biosensors, orange block is association of CE76_-P1_ and hCpn10 and blue is dissociation curve. Data analysed by ForteBio Data Analysis 7.0. (**c**) Inhibition of FVIIa-catalysed activation of FX and amidolytic activity by CE76_-P1_. The IC_50_ value for inhibition of FX activation (▲, blue) by CE76_-P1_ was 1.5 nM. IC_50_ values for inhibition of amidolytic hydrolysis by CE76_-P1_ in the presence (■, red) or absence (●, black) of TF were 6.8 and 8.4 nM, respectively. Inhibition rates of both FXa and amidolytic activity expressed as fractional rate (V_i_/V_0_), and determined by nonlinear regression analysis of the data fit to a four-parameter equation for calculating the IC_50_ and maximal % inhibition. (**d**) Prolongation of PT in human plasma. Prothrombin time (PT) assays performed in a pool of healthy human plasma (n = 5) using Hyland Clotting Analyser. The prolongation of plasma clotting times upon initiation by Thromborel S (Human thromboplastin) and Ca^2+^, measuring the effect of CE76_-P1_ versus controls E-76 peptide and hCpn10.

**Figure 5:**
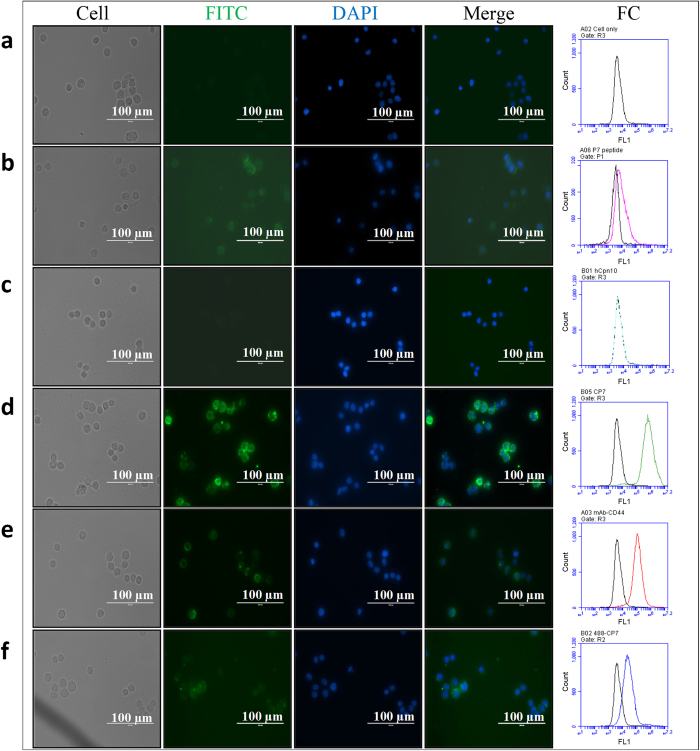
Immunofluorescence and flow cytometry (FC) results for CP7 scaffold binding to native CD44 on MDA-MB-468 breast cancer cells. Immunofluorescence images showing cell surface stain FITC (green), nuclear stain DAPI (blue), and Merge for (**a**) Unstained cells; (**b**) P7 peptide; (**c**) wt hCpn10; (**d**) CP7; (**e**) mouse anti-human CD44 mAb; (**f**) labelled 488-CP7 (1^primary^). Flow cytometry (FC) overlay histograms comparing fluorescence (FL1; 530/30 nm) on cells alone (black) with cells with P7 peptide (magenta), wt hCpn10 (cyan), CP7 (green), anti-CD44 mAb (red) and labelled 488-CP7 (blue).

Structural homologues based on sequences defined in this study (Figs 1 and 2) were created, based on available template X-ray crystal structure of the chaperonin complex from *Thermus thermophilus* (PDB ID. 1WE3). The conformations of the inserted sequences replacing the native mobile loop were modelled as close as possible to the Cpn10 conformation found in 1WE3. This conformation is a free energy minimum for heptameric hCpn10 (Fig. 2). The *N*-terminus modelled as extended chains similarly to the template structure. The inserted E-76 peptide (Fig. 2) was simulated with and without an intact disulphide bond (*Cys23*-*Cys33*).

All models showed heptameric structure of the proteins (Fig. 2), however, for the structural investigation only one monomer was chosen, that is chain “U” of 1WE3 heptamer. This remainder was not part of the actual simulation and figures were prepared after the simulation by superimposing the simulated monomer “U” onto the structure heptamer.

### Molecular dynamics (MD) simulations

MD simulations in this study were performed in conjunction with Computist Bio-Nanotech (Melbourne, Australia) and were based on the NAMD2 program^24^ combined with the CHARMM27 force field^25^. Generalised born implicit solvent (GBIS) method^26^ was applied to increase the conformational sampling time. Approximate dielectric effect of water treated outside of the protein as dielectric continuum solves by the Poisson Boltzmann equation^27^. The van der Waals packing interactions were disregarded in NAMD2’s implementation of the method. The conformational changes that occurred during the MD simulations of examined structures, were reflected as trajectory changes and were processed according to specified geometric descriptors of VMD program^28^. The variations of these trajectories were visually examined.

### Protein Expressions and Purifications

A single colony from freshly grown *E. coli* BL21 (DE3)-pET30a plate was used to inoculate 5 mL TB media, containing 30 μg/mL Kan, shaken at 200 rpm overnight at 37 °C. A dilution (1:100) of overnight culture was used to inoculate 500 mL TB media containing Kan and was incubated at 37 °C, 220 rpm shaking, until OD_600_ reached an approximate 0.6–0.8. Protein expression was induced by adding IPTG to final concentrations of 0.4 mM, and continued for 4 hrs at 26 °C. Cell pellets were harvested by resuspension in a pre-chilled buffer (5 mL/mg), and cells ruptured using a high pressure homogenizer at single pass of 15000 psi. Protease inhibitor cocktail tablet (1/10 mL) was added, then centrifuged at a maximum speed (16000 × g) at 4 °C.

Affinity purification method was first used for capturing *His*-tagged protein. Clarified lysate was loaded onto HisTrap FF crude or HisTrap HP (5 mL column, GE Healthcare) using an ÄKTA prime plus FPLC System (GE Healthcare). The eluted fractions were pooled, concentrated, and buffer exchanged with suitable storage buffer and further purified by gel filtration (GF) chromatography using Superdex 75 gel filtration column (GE Healthcare) using FPLC System of ÄKTA Explorer (Amersham Pharmacia).

### Protein Analyses and Characterisations

A standard method SDS-PAGE (NuPAGE) used for detecting protein expression and purifications, and a SeeBlue Plus2 prestained standard (from Invitrogen) was run in parallel with samples for molecular weight determination. Following electrophoresis, the gel was washed then stained using SimplyBlue SafeStain, and gel images were captured using a Bio-Rad Molecular Imager Gel Doc XR^+^ System and analysed by Image Lab ChemiDoc MP Software.

Protein molecular weights in their native state (in full homogeneity) were investigated using analytical size exclusion-HPLC with a TSK column (TSK G3000SWXL, Tosoh Bioscience) and Agilent 1200 Series HPLC System (Agilent Technologies). Protein samples run in 0.1 M Phosphate (pH 7.8), 0.2 M NaCl in a maximum of 100 μL sample/injection (equivalent to 1 mg/mL) were analysed at a flow rate of 0.8 mL/min (≤70 bar), at 25 °C. A 10 μL BioRad Gel filtration standard (Bio-Rad Laboratories), was applied for determining apparent molecular weight of target proteins from a standard curve of proteins of known size.

### FVIIa binding detections by ELISA

A SpectraMax-M4 plate reader was used to measure the binding of CE76 in a 96-well miroplates. A typical microtitre plate was coated with 100 nM rFVIIa (NovoSeven RT), diluted in PBS, 0.05% (v/v) Tween 20 (PBST, pH 7.4), total of 200 μL per well. The ELISA plate wells were blocked by PBST, 2% (w/v) non-fat dry milk. Purified *His*-tagged CE76 were incubated in various concentrations (from 0.1–1000 nM), and in parallel with *His*-tagged hCpn10 (control), and mAb-mouse anti-FVII human (control). Past incubations (1 hour, 25 °C), plates were washed with PBST then detected by either HRP-conjugated mouse anti-6 × His antibody (1:5000) or HRP-goat anti-mouse antibody (1:5000). A 100 μL of TMB (3,3′,5,5′-Tetramethylbenzidine, Sigma-Aldrich) per well, and terminated after 15 min with 50 μL of 1 M H_2_SO_4_ and measured at 450 nm. Data of blank well (absence of rFVIIa) was used for adjusting background activities and data was fitted by non-linear regression from triplicate independent experiments.

### Protein binding and Kinetic analysis by BLI

Binding affinity and kinetic profile measurements were conducted using Bio-layer Interferometry (BLI) Octet Red instrument (ForteBio). Purified *His*-tagged proteins (1–100 nM) were captured via Ni-NTA biosensors (120–180 sec, at 37 °C, with 1000 rpm), then dipped into multiple concentrations of target proteins (0.1–100 nM) for 180 sec (*k*_*a*_), followed by 10 min dissociation (*k*_*d*_) that was monitored in buffer only. Equilibrium dissociation constant (K_*D*_) of recombinant protein acquired from fitting into 1:1 binding model by global fitting of multiple kinetic traces, and analysed by Data Analysis 7.0 software. A zero nM (control trace) was used for blank run and subtraction.

### FVIIa and FX inhibition Assays

Inhibition of FX activation by TF-VFIIa complex was performed as described^29^. FX activation by TF-FVIIa was determined as a function of CE76_-P1_ (inhibitor) concentrations. A total reaction volume of 100 μl AB containing 175 nM FXa with 10 nM rFVIIa, and 150 nM TF was incorporated into 6 μL TriniCLOT aPTT HS, and incubated with CE76_-P1_ (1 μM, 10-fold dilutions) for 30 min at 37 °C, prior to adding 1 mM S-2765 chromogenic substrate.

Inhibition of amidolytic activity of FVIIa by CE76_-P1_ was determined as a function of inhibitor concentrations, in the presence and absence of TF. Series dilutions of 1 μM (in 10-fold in AB) of CE76_P1_, was incubated with 10 nM rFVIIa and 100 nM TF, for 10–20 min, at 37 °C, prior to adding Chromozym *t*-PA, then measuring the initial reaction velocities at 405 nm by Multiskan GO Microplate Spectrophotometer, at RT. The linear rate of increased absorbance was expressed as fractional activities (V_i_/V_0_), in which the velocity of the substrate hydrolysis in the presence (Vi) or absence (V_0_) of inhibitor CE76_P1_. The relative velocities (V_i_/V_0_) were plotted against the CE76_-P1_ concentration (nM). Controls were made in the absence of CE76_-P1_, rFVIIa, or FX. All experiments were performed in triplicate.

### Plasma clotting time assays

The prothrombin time (PT) and activated Partial Thromboplastin Time (aPTT) assays were performed in pooled citrated-treated human plasma, as described^30^. PT and aPTT assays were triggered by using Thromborel S (human placental thromboplastin) and TriniCLOT aPTT HS (phospholipids mixture) for initiating coagulation of extrinsic and intrinsic cascades, respectively.

PT assay was performed by adding 100 μL citrated plasma to a glass clotting tube and incubated for 10 min at 37 °C. 50 μL CE76 (as inhibitors) were added 0.001–100 μM (10-fold series dilutions) to a final volume of 150 μL. Plasma clotting was triggered by adding Thromborel S, and clotting times were determined. hCpn10, saline and heparin were used as controls.

aPTT assay, was performed similarly to PT assay with sight modifications. A 100 μL citrated plasma, 100 μL TriniCLOT aPTT HS, and CE76 (0.001–100 μM, in 10-fold series dilutions), in glass clotting tubes, with a final volume of 250 μL and incubated for 2 min at 37 °C. Plasma clotting was initiated by adding 50 μL of 2 mM CaCl_2_. Clotting times were determined as a function of protein concentrations at 37 °C in triplicate, using Hyland-Clotek clotting analyser. Time prolongations of both PT and aPTT (in sec) were converted and plotted as folds (T_i_/T_0_).

### Cell lines and cell culture

MDA-MB-468 breast cancer cells overexpressing CD44 were cultured in log-phase growth in 20 mL Gibco RPMI 1640 Medium supplemented with Gibco GlutaMAX with 10% Fetal Bovine Serum (FBS) at 37 °C in a humidified atmosphere (5% CO_2_), in Corning Ultra-Low attachment cell culture flasks (250 mL/25 cm^2^), to a final concentration of 1 × 10^6^ cells/mL.

### Fluorescent conjugation of CP7

CP7 was covalently labelled with fluorophore DyLight 488 (DyLight protein label kit) following the manufacturer’s recommendation.

### Fluorescence Imaging Analysis

Protein binding to cellular CD44 was investigated using fluorescence microscopy (EVOS, Advanced Microscopy Group) by directly adding labelled 488-CP7 to cultured CD44 MDA-MB-468 cells (1 × 10^6^ cells/mL) in 12-well Corning Costar cell culture plates. A 0.1, 0.5, 1, 5, and 10 μM of 488-CP7 per well incubated for 1 hr at 4 °C, and in separate CD44 MDA-MB-468 cells treated with primary antibody FITC-Mouse mAb anti-Human CD44 (1:200 dilution, to final concentration of 5 μg/mL) used as control. Cells were washed with PBS and observed under fluorescence microscope and images were viewed and analysed using Microsoft Office picture manager software.

### Immunofluorescence Microscopy

CD44 expressed MDA-MB-468 cells were treated with CP7 (0.1–10 μg/mL), washed with pre-chilled PBS and then fixed with ice-cold methanol (5 min). Cells were blocked with PBS (pH 7.4), 1% BSA, and 0.1% Triton X-100, for cells permeabilisation and block non-specific interactions. Cells were then incubated with 1–10 μg/mL secondary antibody FITC-Rabbit anti-human Cpn10 (1:1000–1:100 in series dilutions) at 4 °C for 1 hr. MDA-MB-468 cells were treated with 5–10 μg/mL control primary antibody Mouse mAb Human CD44 (1:200 and 1:100 dilution) and then followed by secondary antibody 1 μg/mL Alexa Fluor 488 Rabbit anti-Mouse IgG as control. Similarly cells were treated with 10 μg/mL hCpn10 followed by secondary 5 μg/mL FITC-Rabbit anti-human Cpn10 antibody at 4 °C for 1 hr. Cells were washed and mounted onto microspore slides using VECTASHIELD mounting medium with DAPI for nuclear staining.

In a direct approach, MDA-MB-468 cells were treated with labelled 488-CP7, following the procedures above with slight modifications. Cells were incubated with primary 0.5, 1, 5, and 10 uM 488-CP7 at 4 °C for 1 hr, then washed with pre-chilled PBS, and fixed with ice-cold methanol and blocked with PBS (pH 7.4), 1% BSA, and 0.1% Triton X-100. The control sample was treated with primary antibody FITC-Mouse mAb anti-Human CD44 (1:100 dilution to final concentration of 10 μg/mL). Finally, cells were washed mounted onto microspore slides using VECTASHIELD mounting medium with DAPI. All slides were analysed using EVOS fluorescence microscope.

### Flow Cytometry

Cell surface expression of CD44 were characterised by one-colour immunofluorescent staining using BD Accuri C6 Flow Cytometer System (BD Biosciences). MDA-MB-468 cells were grown as described previously, then resuspended at a concentration of 5 × 10^6^ cells/mL, prior to washing with pre-chilled Flow-cytometry Buffer (FB) of: PBS (pH 7.4), 0.1% Sodium azide, 1% BSA.

For indirect detection approach, a 50 uL aliquot of cells added to equal volume of CP7 (1:2000 and 1:1000) to final concentration of 0.5–1 μg/mL and separately a primary Mouse monoclonal anti-Human CD44 (1:1000, to final concentration of 1 μg/mL), both incubated at 4 °C for 1 hr on rotation. Cells were then washed with equal volume of FB. Cells were then resuspended on a 50 μL FB contained fluorescein isothiocyanate-conjugate secondary antibody; FITC-conjugated Rabbit anti-human Cpn10 (1:2000 dilution) and Alexa Fluor 488-Rabbit anti-Mouse mAb (1:2000 dilution), respectively, and incubated at 4 °C (dark) for 30 min. Cells were then washed and resuspended in 500 μL FB containing 50 μL 4% PFA. Negative controls consisted of grown cells treated identically except for primary CP7 which was replaced with hCpn10, that did not bind to MDA-MB-468 cells when detected with secondary FITC-conjugated Rabbit anti-human Cpn10. Therefore, it was used for subtraction of nonspecific binding and gating.

For direct detection, a 50 uL aliquot of cells were added to equal volume of fluorophore-conjugated of 488-CP7 (1:1000 and 1:500 to final concentration of 1–2 μg/mL) and primary control FITC-mouse anti-human CD44 mAb (1:1000 to final concentration of 1 μg/mL), incubated at 4 °C for 1 hr in dark. Cells were then washed with FB, and then resuspended on 500 μL FB contained 50 μL 4% PFA. Viable cells ≥ 10,000 analysed by using BD Accuri C6 flow cytometer. In this study, excitation was at 495 nm and fluorescence emission was detected by using the 520 nm filter for FITC. Data were analysed using CFlow Sampler software.

## Additional Information

**How to cite this article**: Alsultan, A. M. *et al*. Beyond Antibodies: Development of a Novel Protein Scaffold Based on Human Chaperonin 10. *Sci. Rep.* **6**, 37348; doi: 10.1038/srep37348 (2016).

**Publisher's note:** Springer Nature remains neutral with regard to jurisdictional claims in published maps and institutional affiliations.
